# "Ring out the Old"

**Published:** 1888-12-29

**Authors:** 


### The Hospital

A Weekly Institutional Journal of

**Science, Medicine, Nursing, and Philanthropy.**

*For Hospitals, Asylums, Medical Practitioners, Students, Nurses, Families, Parishes, Congregations, and the Charitable Public.*

Vol. V. No. 118.] [December 29, 1888.

### "Ring Out the Old."

The event of the year 1888 which most filled the mind of the civilised world was the last illness and death of the Emperor Frederick of Germany. Perhaps no monarch known to history ever lived a more kingly life; perhaps no man ever died a more pathetic death. The world is richer by the one, and poorer by the other. He added a distinguished name to the rare list of those whose principles are simple and noble, whose deeds are the fruit of their principles. Those whom Nature made his kin and peers can never cease to mourn, as they can never cease to love him. All good men and women thank God for so pure and manly a life, so patient an endurance of suffering, so calm and trustful a rendering up of honours, duties, and responsibilities into the hand that holds the threads of destiny for kings and peasants alike. Every man has his one life to live, and no more. Happy is he who so lives it that all who have known him may say of him, as they say of the Emperor Frederick, "He lived well; he died as he lived."

In our own country a domestic event of a different kind appealed to the nation's sympathies. The Prince and Princess of Wales celebrated their Silver Wedding. It seems but yesterday that the Princess Alexandra of Denmark left her northern home to live among strangers under a foreign sky. We all awaited her coming with curiosity, not unmixed with anxiety. Her presence allayed both the one and the other. The English people saw in her a true woman, and straightway gave her their hearts. From the allegiance then given they have never wavered, and for the best of all reasons, that they have never had occasion to waver. At this moment, the Princess of Wales, next to the Queen, is the most popular woman in these islands. It was impossible during the anxious months of the Emperor's illness that the Silver Wedding should be celebrated with that unrestrained joyousness which would have been natural to the occasion. But the Prince and Princess did not neglect their public duties because all eyes were not turned exclusively upon them. A Silver Fête was given at South Kensington in aid of the British Home for Incurables. The Princess not only opened the exhibition, but gave the weight of her personal influence steadily to the movement. Somewhat later the Royal pair, with their three Royal daughters, visited the North of London, and amid much enthusiasm, not unmingled with sundry checks of a meteoric character, opened for the inhabitants of that district the new buildings of the Great Northern Central Hospital. Other objects of a charitable character have been indebted to the Prince and Princess for the powerful aid which their presence always gives. Perhaps no Royal pair ever showed more practical interest in public movements of a philanthropic kind than those who now form the connecting link between the Throne and the people of Great Britain.

On the thirteenth of January, 1888, the *Times* announced that the sum of £20,000 required for the foundation of the National Pension Fund for Nurses had been contributed by the munificence of four City merchants, Messrs. Gibbs, Hambro, J. S. Morgan, and Rothschild. In February the Fund was incorporated, and offices were opened for the transaction of business. Recently it was announced that 574 proposals had been received, of which 418 have been completed and the premiums paid, and the remainder are in course of completion. These figures do not represent the whole, as 680 nurses have now joined (see article on another page). The amount received in premiums is £6,145, and the Fund has now in hand invested moneys amounting to over £30,000. This brief record indicates a success probably unparalleled in the history of provident movements. The value of the National Pension Fund to sick nurses it is impossible to exaggerate. It gives to all those who associate themselves with it that feeling of security against sickness and old age which enables them to throw away all anxious care, and to give themselves up with whole-hearted devotion to their arduous and noble work. The year 1888 will not only be memorable in the history of the nursing profession as the year of the founding of the Pension Fund, but also in the history of those merchants and others whose princely generosity and unremitting exertions have made it a concrete fact.

The year has been remarkable for the active part which prominent politicians have taken in philanthropic movements. Lord Randolph Churchill presided at a conference held at the house of Mr. John Aird, M.P., at which it was resolved that Parliamentary boroughs owed a duty to the workless poor of their own constituency; that so far as Paddington was concerned the constituency would deal entirely with the distress within its borders. The movement began and ended in Paddington; but the idea was sound, practical, and entirely successful, and may yet bear fruit in ever-widening areas. Still later Lord Randolph presided at the festival dinner of St. Mary's Hospital, and addressed the assembled philanthropists in language as practical and pointed as it was vigorous and fresh. Previously Lord Rosebery did valuable work by stirring up the public mind in favour of voluntary hospitals, as contrasted with the rate-supported institutions of other countries. These examples it may be hoped will be universally followed by Members of Parliament. Hospitals must be recognised as departments of public service which demand the highest intelligence and the most conscientious care. If Members of both Houses of the Legislature will place themselves at the head of those institutions the result in economy and efficiency will be invaluable.

It is highly satisfactory to find that wealthy men are more and more inclined to consider the case of hospitals in the distribution of their wealth by legacy. In 1888 Mr. Henry Quinn left £50,000 to be disposed of at the discretion of his executors on behalf of the various charities. The largest amount given to any one charity was a sum of £5,000, awarded to the building fund of the Great Northern Central Hospital. That hospital showed its appreciation by naming one of its new and beautiful wards the "Henry Quinn Ward." A still larger sum, amounting to £98,000, was left by Mr. Graham for distribution in a similar way. It is impossible to imagine any objects which are more worthy of the confidence and regard of wealthy benefactors than hospitals. It is true that quite recently certain institutions have been charged with scandalous extravagance; but no charge has been made against the hospital world as a whole. There is no difficulty whatever in finding any number of hospitals, both in London and the country, whose management is all that could be desired. The critics of hospital methods and administration are neither few nor feeble. Miss Louisa Twining a few months ago uttered a note of warning and denunciation against some of the scandals connected with charity bazaars. Miss Twining thinks, and thinks rightly, that good deeds should be done in good ways. She is not among those who hold that the end justifies the means, and who throw over all the minor moralities in order that a good balance at the bank may wind up their proceedings. There is no doubt that honest criticism is productive of much good. Those immediately concerned should welcome it as showing the keen public interest which centres in them and their work.

The cause of charity has progressed in all directions. Whilst we are able to record the adherence of Lord Rosebery and Lord Randolph Churchill to the voluntary principle of hospital support, we point with pride and pleasure to the fact that Mrs. Gladstone, in a letter written at our request, placed before all people the high public duty of supporting these noble institutions. Equally gratifying is it to give permanent record to the progress of this kind of "voluntaryism" among working men. In Leeds the workmen closed Her Majesty's Jubilee year by offering as a free gift two fully-equipped horse ambulances to the Royal Infirmary of that town. In Sunderland, under the able leadership of Mr. A. W. Webster, the working men have contributed large sums, amounting to a very considerable proportion of the whole annual income of their hospital. These examples have stirred up other towns to a healthy rivalry and a generous emulation. Another general movement on behalf of the medical charities, the Hospital Sunday Fund in London, has received more from the congregations than ever before. The Saturday Fund has also held its own, and the management has greatly improved, thanks to Mr. Frewer

In reviewing the course of charitable events during the year, we are led to the conclusion that hospitals are steadily and surely rising in public favour. Never before were they so honoured and trusted as they are now, and never did they merit such confidence so well. Whether we take into consideration their medical practice, their surgical triumphs, their nursing efficiency, or their administrative care, we are alike thankful and glad. Medical charities constitute a world within a world. As in the great world so in the small, there are flaws here and imperfections there. These it is the duty of critics to point out, and of managers to amend. But surveying calmly the hospital world as a whole, we may well say, "These be the noble works of our country; she has created them; she will conserve them; she will make them still more perfect."

